# Hybrid Metasurface Based Tunable Near-Perfect Absorber and Plasmonic Sensor

**DOI:** 10.3390/ma11071091

**Published:** 2018-06-27

**Authors:** Ahmmed A. Rifat, Mohsen Rahmani, Lei Xu, Andrey E. Miroshnichenko

**Affiliations:** 1 Nonlinear Physics Centre, Research School of Physics and Engineering, The Australian National University, Canberra, ACT 2601, Australia; mohsen.rahmani@anu.edu.au; 2 School of Engineering and Information Technology, University of New South Wales, Canberra, ACT 2600, Australia; lei.xu@unsw.edu.au (L.X.); andrey.miroshnichenko@unsw.edu.au (A.E.M.)

**Keywords:** absorber, plasmonics, metasurfaces, optical sensors, nanostructure

## Abstract

We propose a hybrid metasurface-based perfect absorber which shows the near-unity absorbance and facilities to work as a refractive index sensor. We have used the gold mirror to prevent the transmission and used the amorphous silicon (a-Si) nanodisk arrays on top of the gold mirror which helps to excite the surface plasmon by scattering light through it at the normal incident. We numerically investigated the guiding performance. The proposed absorber is polarization independent and shows a maximum absorption of 99.8% at a 932 nm wavelength in the air medium. Considering the real applications, by varying the environments refractive indices from 1.33 to 1.41, the proposed absorber can maintain absorption at more than 99.7%, with a red shift of the resonant wavelength. Due to impedance matching of the electric and magnetic dipoles, the proposed absorber shows near-unity absorbance over the refractive indices range of 1.33 to 1.41, with a zero-reflectance property at a certain wavelength. This feature could be utilized as a plasmonic sensor in detecting the refractive index of the surrounding medium. The proposed plasmonic sensor shows an average sensitivity of 325 nm/RIU and a maximum sensitivity of 350 nm/RIU over the sensing range of 1.33 to 1.41. The proposed metadevice possesses potential applications in solar photovoltaic and photodetectors, as well as in organic and bio-chemical detection.

## 1. Introduction

A perfect absorber is highly desirable for a broad range of applications including for emitters, solar photovoltaic, energy harvesting, light modulation, and sensing [1,2]. The conventional way to achieve maximum absorption is by using the multilayer structures [3,4]. The maximum absorption occurs while the electric permittivity (*ε*) and magnetic permeability (*μ*) satisfy the impedance-matched condition, *ε*(*ω*) = *μ*(*ω*), at the operating frequency [5]. By altering the metal-dielectric layer, perfect absorption can be achieved [4]. However, it leads to the thicker optical device which is in contrast to the current trends of the down scaling integrated optical components resulting in a limited range of applications [1,6]. Metasurface can overcome this problem due to its miniaturized, versatile and fast optical switching capabilities. The dimensions of the metasurfaces are generally far beyond the wavelength, allowing not only the reproducing of the function of bulk optics, but also are capable of manipulating light at nanoscale, which results in easy control of amplitude, polarization and phase of the imposing light and enables a sub-wavelength effect [7,8,9]. Efficient manipulation of light can be realized by all-dielectric metasurfaces; as a result, optically induced electric and magnetic Mie resonance can be achieved [8,10]. By tuning the Mie resonances of metasurfaces, the novel functionalities with tunable and switchable characteristics can be achieved [11]. Due to these unique capabilities, dielectric metasurfaces are the main promising candidates for the perfect absorber. The maximum absorbing performance, polarization independency and wide operating angle are the key parameters that can make an ideal absorber. To date, several efforts have been made to achieve the perfect metasurface-based absorber and various configurations have been reported to realize perfect absorbance with polarization insensitive [12,13], wide-angle [14,15], broadband [1,16], and multiband [17,18] features. Recently, Yijia et al. reported a metamaterial based polarization independent broadband absorber which shows the maximum absorbance of 95% [1]. Four different split-ring resonators (SRRs)-based multiband metasurface absorbers have also been reported, which show a maximum absorbance of 99.9% in the THz region [7]. Qinyu et al. reported a metal-dielectric (Cr-Si) metasurface absorber which can operate in a large incident angle (70 degree) and a wider bandwidth [14]. Another broadband wide-angle polarization-insensitive plasmonic metasurface absorber has been reported with a maximum absorbance of 90% [12]. A bi-directional plasmonic metasurface absorber with free substrate has been reported to extend the absorbing performance for both directions of the incident light [19]. Besides the polarization insensitive, wide angle and broadband performance, the tunable absorbance property is another interesting phenomena in terms of its broad range of applications. Recently, electrically tunable [20] and fluidically tunable [21] metasurface absorber have been reported. However, most of the reported tunable metasurface absorber structures are complex in terms of the fabrication point of view, due to a multilayer and composite design structure. Furthermore, from a practical implementation point of view, the surrounding medium refractive index can change due to unavoidable/uncertain conditions that an absorber performance can hamper. As a result, extensive investigation is needed to observe the surrounding medium effects on absorber performance.

Moreover, to achieve maximum absorbance, plasmonic materials are widely used to diminish the transmission. By achieving the phase matching condition between the metal-dielectric materials, a metasurface absorber can be utilized as a plasmonic sensor as well. By employing this concept, Na et al. reported a plasmonic metasurface-based absorber and a plasmonic sensor that can achieve a maximum absorbance of 99% [15]. Generally, planner plasmonic sensors are required to illuminate light at a certain angle to generate the surface plasmon polariton (SPP) mode [22,23]. However, using the metasurface structure at a normal incident angle also can excite the SPP mode [15]. Recently, Baquedano et al. reported the gold nanobelt grating based plasmonic sensor, which shows the sensing performance at the normal incidence level [24]. Nowadays, plasmonic sensors are being extensively studied due to their extremely sensitive nature in response to the environment. Several number of plasmonic sensors have been reported with different configurations, such as planar waveguide based [23,25], metasurface based [26,27], photonic crystal fiber based [28,29,30,31], optical fiber based [32], etc. However, due to planer structure and normal incidence, such metasurface is a promising candidate for plasmonic sensing. Another important issue of the current plasmonic sensors are the intrinsic loss of the metallic structure. Metallic component has the strong absorption property; as a result zero reflection could be achieved efficiently, providing another novel method to enhance the metasurface based perfect absorber as well as the plasmonic sensor. However, by utilizing the hybrid structure, intrinsic loss could be significantly reduced.

In this work, we proposed a hybrid metasurface absorber with amorphous silicon (a-Si) nanodisks array on top of a gold layer to eliminate the transmission. Silicon nanostructures are CMOS compatible and provide low optical losses compared to plasmonic nanostructures [10]. Our hybrid metasurfaces are designed in such a way to minimize the reflection by following the impedance matching; as a result maximum absorption is achieved. Moreover, the scattered light from the a-Si disks can help to excite the free electrons of plasmonic material gold and generate the localized surface plasmon resonance (LSPR). This unique feature makes our proposed device applicable for the refractive index sensing, as well. By changing the refractive index of the surrounding medium, resonance conditions can be tuned and this feature can be used to detect unknown analytes.

## 2. Structural Design and Numerical Analysis

**Figure 1:**
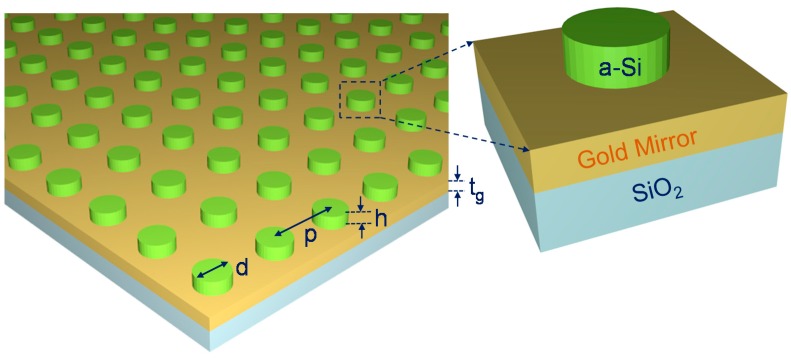
Schematic of the proposed metasurface device (inset shows the unit cell of the metasurface).

Figure 1 shows the proposed hybrid metasurface device and inset figure shows the unit cell of the metasurface. SiO_2_ has been used as a substrate and experimental data has been used to define its optical properties. A thick gold layer (t_g_ = 80 nm) is used on top of the substrate, which is larger than the skin depth. As a result, the penetration of the electromagnetic wave will be negligible in the near infrared region and the transmission will be minimal. The a-Si nanodiks arrays are arranged on top of the gold layer. The diameter of the disk is d = 230 nm, thickness is h = 50 nm and the array of disks are arranged with periodicity, p = 670 nm.

The a-Si disks are arranged in a symmetric way as a result proposed device will facilitate the polarization insensitive performance which is one of the key requirements for an ideal absorber. We have used the experimental refractive index (RI) value of a-Si which will enhance the performance accuracy in terms of practical realization. The gold film properties are adopted from Johnson and Christy model [33].

To observe the guiding performance of the proposed hybrid metasurface device, we used the commercially available simulation software CST Microwave studio with the unit cell boundary condition. The waveguide ports have been used to investigate the scattering responses of the device.

## 3. Results and Discussions

### 3.1. Performance Analysis with Respect to Perfect Absorber

**Figure 2:**
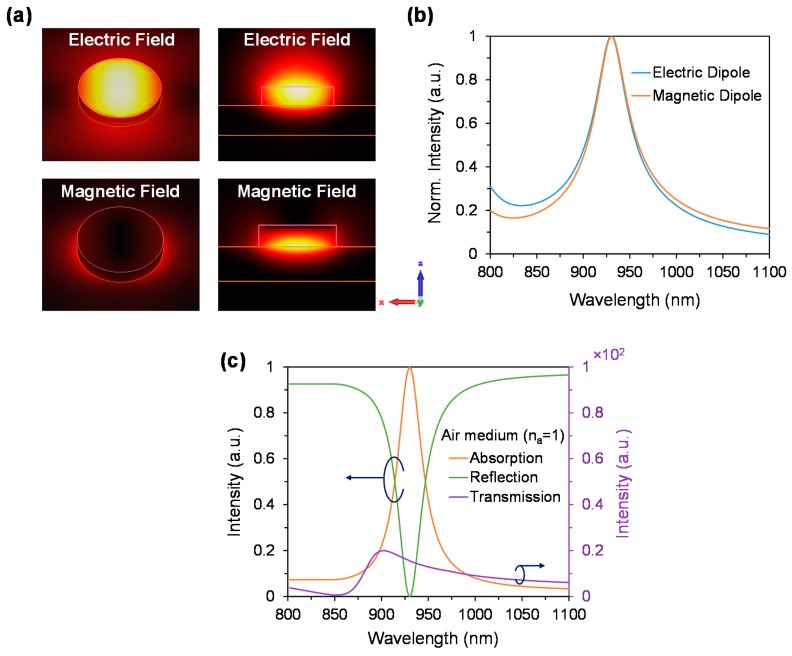
(**a**) Electric and magnetic fields distribution of the proposed metasurface absorber in air superstrate. The nanodisk diameter is d = 230 nm; thickness, h = 50 nm and period, p = 670 nm. (**b**) Resonant overlap of electric and magnetic dipoles, resulting in impedance matching phenomenon, and (**c**) Absorption, reflection and transmission spectra of the proposed metasurface in air-medium (n_a_ = 1).

The incident electromagnetic wave (EM) is polarized along the x-axes. The electric and magnetic fields distribution of the proposed dielectric absorber are shown in Figure 2a,b respectively. It is worth noting that electric field distribution is stronger inside the disk and on top of the disk, while the magnetic field is more stronger beneath the disk (metal/dielectric interface) and surrounding of the disk [34,35]. In the proposed device, the suppression of the reflectance occurs due to the perfect matching of the electric dipole and magnetic dipoles (Figure 2b), which is also known as impedance matching.

In the proposed device impedance matching occurs at 930 nm wavelength. The minimal reflectance leads to the maximum absorption. The general Equation to calculate the absorption is as following,

Absorption (A) = 1 − Transmission (T) − Reflection (R)(1)

Due to thick gold layer, the transmission of the proposed device will be negligible and as a result, the absorption directly depends on the reflection. The relationship of absorption, reflection and transmission spectra with respect to wavelength is shown in Figure 2c. As can be seen in Figure 2c, the transmittance and reflectance are minimal at the operating wavelength of 930 nm, resulting in a maximum absorption of 99.8% is achieved. This absorbing performance is observed in the ideal case (air medium), however by changing the outside/environment refractive index (n_a_) of the device, it is possible to tune the impedance matching wavelength. As a result, by changing the refractive index of the environment, the operating wavelength also could be significantly tuned.

**Figure 3:**
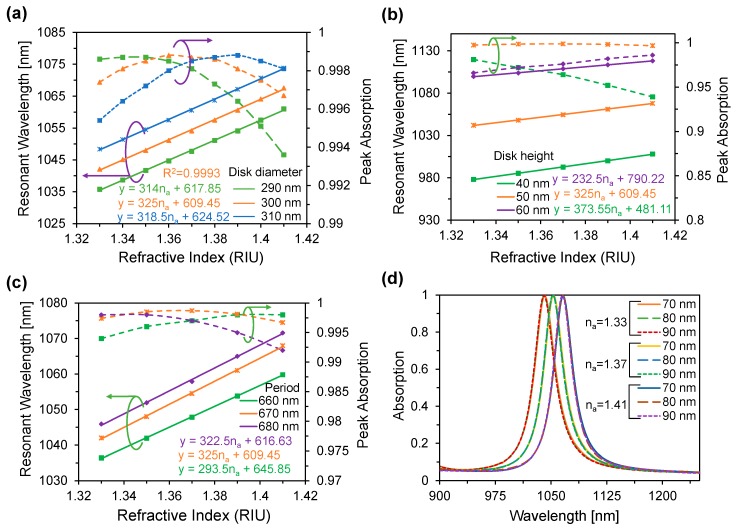
Resonant wavelength as a function of refractive index with varying the (**a**) disk diameter, (**b**) disk height and (**c**) period. (**d**) Absorption spectra with varying gold thickness and analyte RI.

To ensure the maximum absorbing and the sensing performance over the broad refractive index range, we have optimized the disk diameter, disk height, period and the gold thickness shown in Figure 3. We have found the disk diameters 290, 300 and 310 nm show the good absorption over the analyte refractive index range of 1.33 to 1.41, shown in Figure 3a. However, among these diameters, the proposed metasurface absorber exhibits the maximum average absorbance at the disk diameter 300 nm over the analyte RI range of 1.33 to 1.41. Moreover, it also shows the maximum average sensitivity of the proposed sensor is 325 nm/RIU and the linear fitting of the resonant wavelength R^2^ = 0.9993 while d = 300 nm. As a result, we optimized the disk diameter 300 nm of our proposed device to further investigate the tunable absorbance performance.

Figure 3b shows the effects of disk height on the absorption and the sensor performance. It can be seen that, maximum average sensitivity 373.55 nm/RIU is achieved for h = 60 nm, however, the absorption response is diminish over the refractive index range of 1.33 to 1.41. On the contrary, at h = 50 nm, it shows the maximum average absorption and also the moderate average sensitivity as a result the disk-thickness has been optimized to 50 nm.

The center-to-center distance between the two adjacent disks corresponds to period. Its effect was also investigated on the device performance (see Figure 3c). With the increase of period, resonant wavelength shows the red-shift and absorption also increase initially. However, considering the average absorption and the sensing response, the period of the proposed device has been optimized to 670 nm. To diminish the transmission of the proposed device, a thicker gold layer has been used which acts as a mirror. According to Figure 3d, it can be seen that by varying the gold thickness, the resonant wavelength remains the same for the specific analyte RI, which indicates that gold thickness has negligible influence on the device performance as long as it is larger than the skin depth.

**Figure 4:**
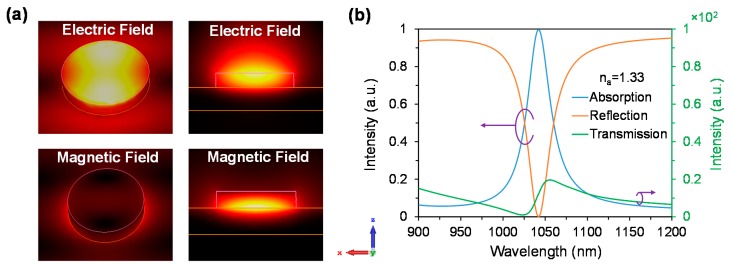
(**a**) Electric and magnetic fields distribution of the proposed metasurface absorber for n_a_ = 1.33. The nanodisk parameters diameter is d = 300 nm; thickness, h = 50 nm and period, p = 670 nm, and (**b**) Absorption, reflection and transmission spectra of the proposed metasurface absorber at medium (n_a_ = 1.33).

**Figure 5:**
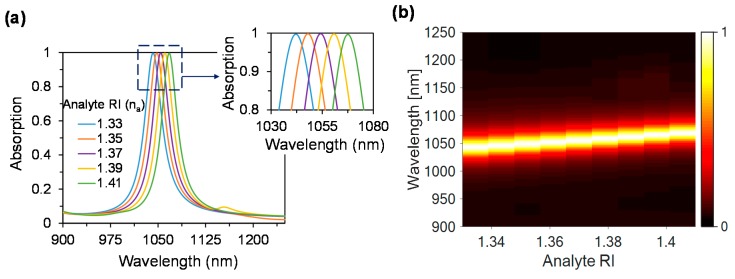
(**a**) Absorption spectra for various analytes from 1.33 to 1.41, and (**b**) Full 2D representation.

Figure 4a shows the electric and magnetic field distribution at n_a_ = 1.33, which is in good agreement with n_a_ = 1 where the magnetic field is much stronger beneath the disk and surrounding the disk. Due to change of RI n_a_ = 1.33 the operating wavelength is shifted towards the larger wavelength of 1042 nm, shown in Figure 4b. The maximum absorption of 99.7% is achieved at n_a_ = 1.33. With the increase of analyte refractive indices from 1.33 to 1.41, the impedance matching condition shifts towards the longer wavelength, which results in a redshift of the operating wavelength (see Figure 5a). The maximum achieved absorption is 99.7%, 99.9%, 99.9%, 99.8% and 99.7% at the resonant wavelengths of 1042, 1048, 1054, 1061 and 1068 nm, respectively, while the analyte RI are 1.33, 1.35, 1.37, 1.39 and 1.41, accordingly.

Figure 5b shows the absorption as a function of analyte refractive index where it is clearly visible that with the increase of analyte RI, the operating wavelength shows the redshift. It is noticeable that by varying the environment refractive indices from 1.33 to 1.41, the proposed dielectric metasurface absorber shows an average absorbance of 99.8%, which is a good indication for using the proposed device as a tunable absorber.

**Figure 6:**
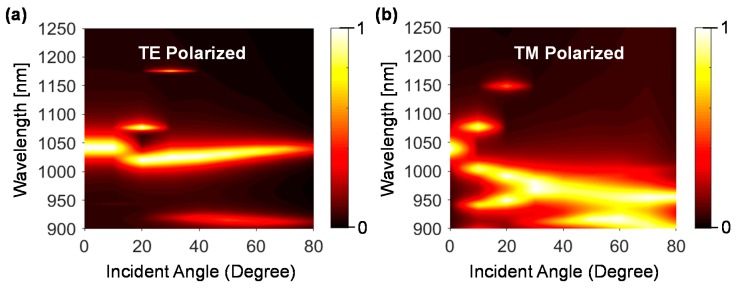
Angular dispersions of the absorbance peak for (**a**) TE and (**b**) TM polarized mode.

Like the tunable absorption performance, polarization insensitive and incident angle independency are important parameters. Due to the symmetric properties of dielectric disks, the proposed device will ensure the polarization insensitive performance at the normal incident level. Figure 6 shows the proposed metasurface absorber performance in terms of incident angle. According to Figure 6a, for TE polarization, with the increase of incident angle, the absorbance level reduces gradually, however, it maintains a constant absorption level above 94% until 50 degrees and then is reduced to 42% at 80 degrees. It indicates that the electric field direction remains unaffected until the incident angle of 50 degrees. Although the operating wavelength is slightly reduced at 15 degrees, it remains almost same wavelength over the wide incident angle of 0–80 degrees. For TM polarization, the proposed device is maintained above a 90% absorbance over the incident angle of 0–80 degrees. However, for both TE and TM mode, the proposed sensor maintains the same operating wavelength 1040 nm up to the incident angle of 8 degrees.

### 3.2. Performance Analysis with Respect to Plasmonic Sensor

**Figure 7:**
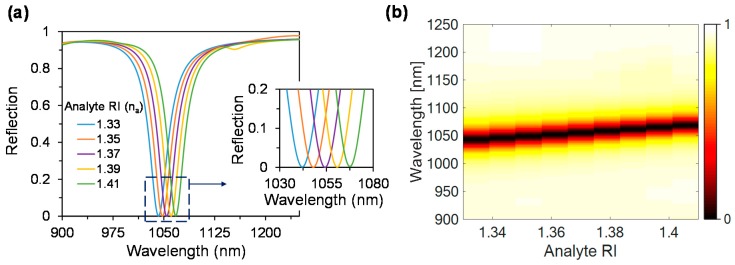
(**a**) Reflection spectra with varying the analyte RI from 1.33 to 1.41, and (**b**) Reflection intensity as a function of analyte RI.

The plasmonic sensing feature of the proposed dielectric metasurface device is shown in Figure 7. The zero-reflectance achieved due the perfect impedance matching. The proposed device shows the localized surface plasmon polariton mode which propagates along the metal-dielectric interface at the zero-incident angle shown in Figure 4a. The sensing mechanism relies on the resonant wavelength shift which occurs due to the change of the refractive index of the surrounding medium. Due to the change of refractive index from 1.33 to 1.41, the proposed sensor shows the red-shift of the resonant wavelength (see Figure 7a). Figure 7b also clearly indicates that the zero-reflectance position is changing towards the larger wavelength with the analyte RI varying from 1.33 to 1.41. Due to the refractive indices of 1.33, 1.35, 1.37, 1.39 and 1.41 resonant wavelengths shifted to 1042, 1048, 1054, 1061 and 1068 nm, respectively. By measuring the resonant wavelength shift sensitivity of the proposed sensor can be calculated by the following equation [36],

(2) $$\mathrm{Sensitivity},\;s_{\lambda }(\lambda )=\frac{{\Delta \lambda }_{\mathrm{peak}}}{{\Delta n}_{a}}$$

where Δλ_peak_ is the difference between two resonant wavelengths and Δn_a_ is the analyte refractive index difference.

Using the wavelength interrogation method, the proposed sensor shows the sensitivity of 300, 300, 350, 350 and 350 nm/RIU for analyte RI of 1.33, 1.35, 1.37, 1.39 and 1.41, respectively.

The resolution of the sensor is another important parameter that describes how small variation of analyte RI can be detected [36],

(3) $$\mathrm{Resolution},\;R\;=\frac{{\Delta n}_{a}\times {\Delta \lambda }_{\min}}{{\Delta \lambda }_{\mathrm{peak}}}\;\mathrm{RIU}$$

where Δλ_min_ is the minimum spectral resolution. Considering the minimum 1% detection capability of the proposed sensor, it shows the maximum sensor resolution of 2.9 × 10^−4^ RIU. This indicates that the proposed sensor is able to detect the 10^−4^ scaled smallest RI changes of the samples.

**Figure 8:**
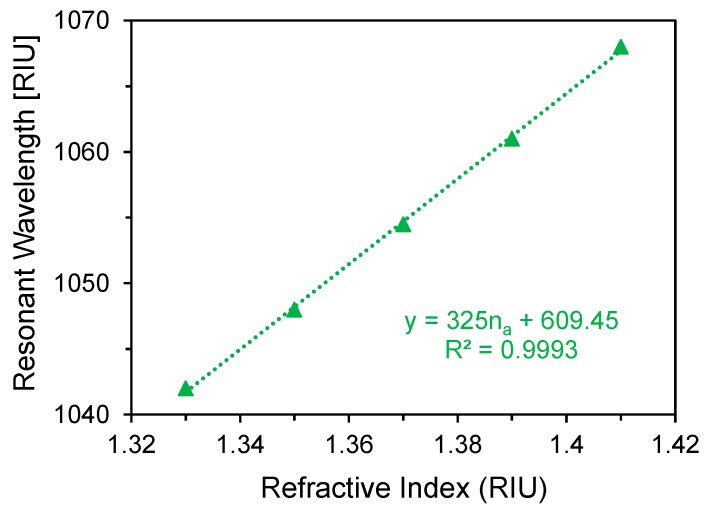
Resonant wavelength as a function of refractive index with the optimum parameters.

The linear fitting of the proposed sensor with the optimum parameters are shown in Figure 8.

The regression equation for the proposed sensor is, y(nm) = 325n_a_ + 609.45 for 1.33 ≤ n_a_ ≤ 1.41, where y is the resonant wavelengths of the analyte in nanometer and n_a_ is the analyte RI. From Figure 8, the average sensitivity of the proposed sensor is 325 nm/RIU and R^2^ value is 0.9993, indicating the good fitting of the sensor response. Owing to its high sensitivity and linearity, the proposed metasurface based plasmonic sensor could be implemented as a standardized sensor for refractive index sensing.

In terms of practical realization, proposed device could be easily fabricated by following the standard electron beam lithography (EBL) process and due to planar surface the gold layer could be achieved by electron beam evaporation (E-beam) process [37]. The proposed plasmonic sensor works at zero incident angle which makes it more promising compared to the conventional prism based plasmonic sensors where oblique incident is required to achieve the plasmonic phenomena. Furthermore, due to zero incident angle, characterization of the proposed device will be straight forward.

## 4. Conclusions

In this work, we numerically investigated a hybrid metadevice as a perfect absorber and a plasmonic sensor, simultaneously. We investigated the absorbing performance in the ideal case (air-medium) and also changing the surrounding medium refractive index which gives the tuning feature of the proposed absorber. It shows the maximum absorbance of 99.8% in the ideal case and the average absorbance of 99.7% over the surrounding medium refractive index variation from 1.33 to 1.41. The proposed plasmonic sensor also shows the average sensitivity of 325 nm/RIU with linearity of 0.9993 over the sensing range of 1.33 to 1.41. Alongside this, it exhibits the maximum sensor resolution of 2.9 × 10^−4^ RIU. By combing the device performance and straight forward practical implementation process, proposed metadevice is a suitable candidate as an ideal perfect absorber as well as the plasmonic sensor.
